# Self-Guided Molecular Simulation Methods

**DOI:** 10.3390/ijms262110410

**Published:** 2025-10-26

**Authors:** Xiongwu Wu, Bernard R. Brooks

**Affiliations:** Laboratory of Computational Biology, National Heart, Lung, and Blood Institute (NHLBI), National Institutes of Health (NIH), Bethesda, MD 20892, USA; brb@nih.gov

**Keywords:** molecular simulation, molecular dynamics, Langevin dynamics, conformation sampling, conformation search, self-guided molecular dynamics, self-guided Langevin dynamics, replica-exchanging

## Abstract

This work reviews self-guided (SG) molecular simulation methods and illustrates the characteristics and applications of these methods through several example simulations. The main characteristic of SG methods is that past motion in simulations is used to guide future motion. Two forms of these methods are self-guided molecular dynamics (SGMD) and self-guided Langevin dynamics (SGLD). SG methods achieve an enhanced conformational search through promoting low-frequency motion. A simple local averaging scheme is used to extract low-frequency properties from past simulation trajectories to promote low-frequency motion, which significantly enhances conformational search efficiency with little overhead in computing cost. Based on a generalized Langevin equation (GLE), an SGLD-GLE simulation method is developed, which has enhanced conformational searching ability and at the same time can vigorously sample the canonical ensemble. A reformulation of the SG methods leads to a quantitative relation between the guiding parameters and the conformational distribution, which allows the SG methods to be combined with the replica exchange scheme to perform replica-exchanging self-guided simulations (RXSGMD/RXSGLD). RXSGMD/RXSGLD are much more efficient than temperature-based replica exchange methods, especially for large systems.

## 1. Introduction

Molecular dynamics simulation is carried out by letting atoms move according to Newton’s equation of motion. The energy landscape on which atoms move is highly rugged, which leads to many motion modes. High-frequency motions, such as bond stretching and bending, dominate thermal motion in macromolecular systems. However, it is low-frequency motions that lead to many interesting conformational changes, such as protein folding and ligand binding. High-frequency motions limit the size of simulation time steps, meaning that expensive energy calculations must be performed many times before the desired conformational change can be achieved.

Most enhanced conformational sampling methods rely on user-specified biased potentials to help overcome energy barriers, such as metadynamics [1], accelerated molecular dynamics [2], and self-adapted accelerated molecular dynamics [3]. Self-guided (SG) molecular simulation methods include self-guided molecular dynamics (SGMD) [4,5] and self-guided Langevin dynamics (SGLD) [6,7,8,9,10], achieve efficient conformational searching and sampling based on enhancing low-frequency motions. Originally, SGMDs incorporated local average forces into the equation of motion and SGLD used local average momentum to guide atomic motion to achieve an enhanced conformational search. SG methods are as robust as normal molecular dynamics or Langevin dynamics simulation methods. Users do not need to input system-specific information such as where energy barriers are or what degrees of freedom should be focused on. Instead, they enable systems to get out of local minimums through promoting low-frequency motion. Simulated annealing methods [11,12,13] have been used to overcome energy barriers. However, rising temperature will change conformational distribution dramatically, causing thermal expansion and melting and other undesired perturbations to a simulation system. Low-frequency momentum is related to diffusional motion, which is effective in enhancing diffusion-controlled search. Low-frequency force is related to a smoothed energy surface, which is effective in enhancing energy barrier-controlled search. These two guiding forces can be utilized individually or together to provide the desired enhancement in conformational search. SG methods propel atoms to perform a self-guided motion which does not incur significant overheads in computing costs. It should be noted that SG methods are applied on an atom-by-atom basis. And if all atoms are included in the enhancement, then all low-frequency motion will be enhanced. But it is also possible to have some atoms with less or no enhancement, where the latter would mean simply combining an SG method with ordinary molecular dynamics in the same run. Many studies of long time-scale events such as peptide folding [14,15,16,17], conformational reorganization [18], conformational state recognition [19], and conformational transitions [20,21,22] have been achieved using SG methods.

Compared to many simulation methods developed for enhanced sampling, SG methods do not involve extra calculation in potential energies, which is the most expensive part of molecular simulations, and do not need knowledge of energy barriers. Therefore, SG simulations are similar to conventional simulations in terms of setting up, running, and analyzing. The enhancement is controlled by the guiding parameters that enable simulation systems to undergo enhanced low-frequency motion without raising temperature. Comparisons with high-temperature simulations and replica-exchanging molecular dynamics simulations are provided in the method development papers [4,5,6,7,8,9,10].

The evolvement of the SG simulation methods comes with the progress in understanding guiding effects on molecular systems. In the earliest version of SGMD [4,5], the guiding forces were local average nonbonded forces, with the intention of accelerating conformational search in the free energy surface. This method led to successful simulations of peptide folding [14,15,16,17,23], ligand docking [24], and liquid crystallization [4]. Based on Langevin dynamics, a relation between local average forces and local average momenta was established. Local average momentum was found to be an effective guiding force to increase sampling efficiency in Langevin dynamics, which led to the SGLD method [6]. In our early attempt to quantitatively describe the conformational distribution of the SG methods, we separated properties of molecular systems based on frequency through the local averaging scheme and derived the partition function of the self-guided ensemble. This self-guided ensemble partition function allowed us to develop the force–momentum-based self-guided Langevin dynamics, or SGLDfp [25], and the replica-exchanging self-guided Langevin dynamics, RXSGLD [26]. The physical basis of SGLD was further explored through the generalized Langevin equation (GLE). An equation of self-guided motion was rigorously derived by a specific choice of the memory kernel, which lead to the SGLD-GLE method [9]. The SGLD-GLE method maintains the canonical ensemble, and at the same time enhances conformational sampling efficiency. Most recently, by reformulating the equation of Newtonian motion to the equation of Langevin motion, we generalized the self-guided motion, which led to an understanding of the two guiding factors and derived a relation between their effects and conformational distribution [10]. This generalized self-guided simulation method is recommended for SGMD/SGLD simulations.

In this work, we are going to present SG methods in the most recent version, the generalized SGMD/SGLD method, followed by a replica-exchange version, RXSGMD/RXSGLD. A generalized Langevin equation-based SGLD method, SGLD-GLE, is also presented for its rigorous theoretic basis. The SG methods presented here can be found in the current releases of CHARMM (c49b1 and later version) [27] and Amber (2024 and later version) [28].

## 2. Methods and Algorithms

### 2.1. The Equation of Self-Guided Motion

The core of the SG methods is the equation of self-guided motion. The most recent reformulation of the SG method leads to the following equation of generalized self-guided motion [10]:

(1) $$\dot{p}=F+\lambda \xi \tilde{p}+\mu (\tilde{F}-\tilde{\tilde{F}})$$

Here, **p** denotes the atom’s momentum and F denotes the apparent force acting on the atom, including all contributions such as molecular interactions, constraint forces from SHAKE [29] if present, friction forces and random forces in Langevin dynamics, velocity scaling for constant temperature simulations, etc. The left side of Equation (1) is the time derivative of momentum, $\dot{p}$, and the right side is the total force, including the momentum guiding force $\lambda \xi \tilde{p}$, and the force guiding force $\mu (\tilde{F}-\tilde{\tilde{F}})$.

The cap “~” on a property represents its local average, which is calculated during a simulation using the following scheme along trajectories [4,5,6]:

(2) $$\tilde{P}\left(t\right)=\frac{1}{t_{L}}\int_{-\infty }^{t}{Pe}^{-\frac{t-\tau }{t_{L}}}d\tau \approx \left(1-\frac{\delta t}{t_{L}}\right)\tilde{P}\left(t-\delta t\right)+\frac{\delta t}{t_{L}}P(t)$$

Here, P denotes any time-dependent property and $\tilde{P}$ is its local time average. A parameter, $t_{L}$, is introduced to denote the local average time, which is related to a frequency threshold, $1/t_{L}$. A local average calculated with Equation (2) will filter down the high-frequency portion and keep the low-frequency portion related to the frequency threshold.

The guiding forces are local averages of momentums or forces and are scaled by the momentum guiding factor λ and the force guiding factor μ, respectively. The low-frequency characteristics of the guiding forces provide a way to alter the low-frequency motion of the simulation system. In molecular systems, atoms interact with their surroundings in the form of bonded and nonbonded interactions. These interactions act as a thermal bath, producing frictions to atom movement. In SG methods, momentum guiding forces are intended to reduce these frictions in the direction of low-frequency motion. These friction effects can be quantified with an apparent friction constant, ξ, which is estimated during simulation:

(3) $$\xi =-\frac{<\left(\tilde{F}-\tilde{\tilde{F}}\right)\cdot \tilde{p}>}{<\tilde{p}\cdot \tilde{p}>}$$

From Equation (3) we can see that the apparent friction constant is a conversion factor between the local average of momentum, $\tilde{p}$, and the local average of force deviation, $\tilde{F}-\tilde{\tilde{F}}$, in the direction of $\tilde{p}$. Both guiding forces can influence low-frequency motion. When λ>0,  the momentum guiding force promotes diffusion-controlled conformational search, and when μ<0, the force guiding force promotes energy barrier-controlled conformational search. The two guiding parameters can be converted from each other by the following relation:

(4) $$\lambda_{\mu }={\left(1+\mu \right)}^{2}-\frac{1}{1+\mu }$$

We call $\lambda_{\mu }$ the balanced momentum guiding factor of μ. From Equation (4) we can derive the balanced force guiding factor, $\mu_{\lambda }$, of λ:

(5) $$\mu_{\lambda }=\frac{2^{1/3}\lambda }{{\left(27-3\sqrt{81-12\lambda^{3}}\right)}^{1/3}}+\frac{{\left(27-3\sqrt{81-12\lambda^{3}}\right)}^{1/3}}{2^{1/3}3}-1$$

### 2.2. Conformational Distribution of SGMD/SGLD

Based on an energy–force relation, we can reasonably claim that the guiding force $\mu (\tilde{F}-\tilde{\tilde{F}})$ corresponds to an energy surface $\mu \left({\tilde{E}}_{p}-{\tilde{\tilde{E}}}_{p}\right)$. For the momentum guiding force $\lambda \xi \tilde{p}$, based on the balanced force guiding factor, $\mu_{\lambda }$, we can claim that it corresponds to an energy surface $-\mu_{\lambda }\left({\tilde{E}}_{p}-{\tilde{\tilde{E}}}_{p}\right)$. Therefore, for a SG simulation with the guiding factors, λ and μ, the corresponding SG potential energy surface is $\left(\mu -\mu_{\lambda }\right)\left({\tilde{E}}_{p}-{\tilde{\tilde{E}}}_{p}\right)$. The partition function on this potential energy surface has the following form:

(6) $$Q_{SG}(\lambda ,\mu ,\beta )=\Omega \;\exp\left(-\beta \left(E_{p}+\left(\mu -\mu_{\lambda }\right)\left({\tilde{E}}_{p}-{\tilde{\tilde{E}}}_{p}\right)\right)\right)$$

Here, $\beta =\frac{1}{kT}$ and k is the Boltzmann constant. Equation (6) represents a SG ensemble, which describes the conformational distribution from a SG simulation. Canonical ensemble averages of any property can be calculated in a SG simulation through the following reweighting scheme:

(7) $$<P>=\frac{\sum_{i}w_{t}\left(\lambda ,\mu ,\beta \right)P_{t}}{\sum_{i}w_{t}\left(\lambda ,\mu ,\beta \right)}$$

The summations in Equation (7) run over all sampled conformations. The subscript *t* represents quantities at time frame *t*. For convenience, we drop subscript *t* in all following discussions. The conformation weight, $w\left(\lambda ,\mu ,\beta \right)$, is given as follows:

(8) $$w\left(\lambda ,\mu ,\beta \right)=\exp\left(-\beta \left(\mu -\mu_{\lambda }\right)\left({\tilde{E}}_{p}-{\tilde{\tilde{E}}}_{p}\right)\right)$$

When $\mu =\mu_{\lambda }$, or $\lambda =\lambda_{\mu }$, Equation (8) shows that $w\left(\lambda ,\mu ,\beta \right)=1$ and a SG simulation will sample the canonical ensemble.

### 2.3. A Leap-Frog Algorithm for SGMD/SGLD

We implemented the SGMD/SGLD simulation method in two widely used molecular modelling and simulation packages, CHARMM [27,30] and AMBER [28]. Implementation of the SG methods involves only the integration of the equation of motion and leaves the most expensive energy calculation intact. The SG methods are just like conventional molecular dynamics (MD) or Langevin dynamics (LD) in setting up, performing, and analyzing results. The initial velocities are typically generated randomly based on simulation temperatures, just like in MD or LD. Therefore, SG simulations have very little overheads in computing cost per time step compared to conventional simulations. The leap-frog algorithm is widely used in simulation packages to integrate the equation of motion. Here, we present a leap-frog algorithm to integrate the equation of the SG motion.

The equation of the SG motion, Equation (1), needs several local average properties for each atom. We find many local average properties are related to the local average positions, $\tilde{r}$, and their local averages, $\tilde{\tilde{r}}$ [10]. Therefore, we only need arrays to store $\tilde{r}$, $\tilde{\tilde{r}}$, and $\tilde{p}$, and can use these arrays to calculate all other local average properties. In this algorithm, atoms are integrated independently of other atoms, which saves inter-process communication and benefits parallel computing.

(1) At current the time step t, the potential energy, $E_{p}$, and forces, f, which include the random forces in Langevin dynamics, are calculated.

(2) Use $E_{p}$ and r to calculate local average properties.

Local average energies:

(9) $${\tilde{E}}_{p}(t)=\left(1-\frac{\delta t}{t_{L}}\right){\tilde{E}}_{p}\left(t-\delta t\right)+\frac{\delta t}{t_{L}}E_{p}\left(t\right)$$

(10) $${\tilde{\tilde{E}}}_{p}(t)=\left(1-\frac{\delta t}{t_{L}}\right){\tilde{\tilde{E}}}_{p}\left(t-\delta t\right)+\frac{\delta t}{t_{L}}{\tilde{E}}_{p}\left(t\right)$$

We can calculate the reweighting factor, $w\left(\lambda ,\mu ,\beta \right)$, of the current conformation from ${\tilde{E}}_{p}$ and ${\tilde{\tilde{E}}}_{p}$, according to Equation (8).

Local average positions:

(11) $$\tilde{r}\left(t\right)=\left(1-\frac{\delta t}{t_{L}}\right)\tilde{r}\left(t-\delta t\right)+\frac{\delta t}{t_{L}}r\left(t\right)$$

(12) $$\tilde{\tilde{r}}\left(t\right)=\left(1-\frac{\delta t}{t_{L}}\right)\tilde{\tilde{r}}\left(t-\delta t\right)+\frac{\delta t}{t_{L}}\tilde{r}\left(t\right)$$

From $\tilde{r}\left(t\right)$ and $\tilde{\tilde{r}}\left(t\right)$, other local average properties, $\tilde{p}$, $\tilde{F}$, and $\tilde{\tilde{F}}$ are calculated:

(13) $$\tilde{p}\left(t\right)=\frac{m}{t_{L}}(r\left(t\right)-\tilde{r}\left(t\right))$$

(14) $$p\left(t\right)=\tilde{p}\left(t-\delta t\right)+\frac{t_{L}}{\delta t}(\tilde{p}\left(t\right)-\tilde{p}\left(t-\delta t\right))$$

(15) $$\tilde{F}\left(t\right)=\frac{1}{t_{L}}p\left(t\right)-\frac{m}{t_{L}^{2}}(r\left(t\right)-\tilde{r}\left(t\right))$$

(16) $$\tilde{\tilde{F}}\left(t\right)=\frac{m}{t_{L}^{2}}(r\left(t\right)-2\tilde{r}\left(t\right)+\tilde{\tilde{r}}\left(t\right))$$

According to Equations (13)–(16), only local average positions, $\tilde{r}\left(t\right)$ and $\tilde{\tilde{r}}\left(t\right)$, need to be stored during a SG simulation, and, $\tilde{p}$, $\tilde{F}$, and $\tilde{\tilde{F}}$ can be calculated as needed.

(3) Calculate apparent friction constants.

The apparent friction constants are needed to convert momenta to forces. According to Equation (6), ensemble averages of $\left(\tilde{F}-\tilde{\tilde{F}}\right)\cdot \tilde{p}$ and $\tilde{p}\cdot \tilde{p}$ are needed to calculate the apparent friction constant of each atom. These ensemble averages are replaced with long-time local averages so we can calculate atomic apparent friction constants on the fly. We use an average time, $t_{avg}$, typically 10 times the local average time, $t_{L}$, for long-time average calculation. The long-time averages are stored in two scalar arrays, FP and PP.

(17) $$FP(t)=\left(1-\frac{\delta t}{t_{avg}}\right)FP(t-\delta t)+\frac{\delta t}{t_{avg}}\left(\tilde{F}-\tilde{\tilde{F}}\right)\cdot \tilde{p}$$

(18) $$PP(t)=\left(1-\frac{\delta t}{t_{avg}}\right)PP(t-\delta t)+\frac{\delta t}{t_{avg}}\tilde{p}\cdot \tilde{p}$$

(19) $$\xi =-\frac{FP(t)}{PP(t)}$$

Equations (17)–(19) allow the apparent friction constants for each atom to be calculated during simulations. The long-time local averages fluctuate around ensemble averages, as do the apparent friction constants. Even though at any moment the long-time local averages could deviate from the ensemble averages, the overall effects are believed to resemble a result from accurate ensemble averages. Right now, it is difficult to provide theoretical support for this idea, but the reweighting results presented in the result Section 3.1 validate this approximation. Alternatively, one can input the apparent friction constants of atoms calculated from previous simulations at the beginning of a simulation and use them in SG simulations of identical guiding parameters.

(4) Calculate the guiding force for each atom:

(20) $$g=\mu (\tilde{F}-\tilde{\tilde{F}})+\lambda \xi \tilde{p}$$

(5) Calculate the energy conservation scaling factor:

The guiding force will do work to the simulation system. To cancel energy input due to the guiding force, an energy conservation scaling factor, η, is used to maintain energy conservation. The energy conservation scaling factors are atom-specific, and their calculation needs no information from other atoms; therefore, it is efficient in parallel computing.

(21) $$\eta =\frac{\left(2+\gamma \delta t\right)g\cdot p_{0}}{{2p}_{0}^{2}-g\cdot p_{0}\delta t}$$

Here, γ is the friction constant for SGLD, and for SGMD γ=0. $p_{0}$ is the free move momentum at time *t*.

(22) $$p_{0}=mv\left(t-\frac{\delta t}{2}\right)+\left(f+g-\gamma mv\left(t-\frac{\delta t}{2}\right)\right)\frac{\delta t}{2}.$$

(6) Forward velocities to $t+\frac{\delta t}{2}$:

(23) $$v\left(t+\frac{\delta t}{2}\right)=\frac{\left(1-\frac{(\gamma +\eta )\delta t}{2}\right)}{1+\frac{(\gamma +\eta )\delta t}{2}}v\left(t-\frac{\delta t}{2}\right)+\frac{f+g}{1+\frac{(\gamma +\eta )\delta t}{2}}\frac{\delta t}{m}$$

For SGLD, **f** is the interaction force plus the random force, and for SGMD, there is no random force in **f**.

(7) Forward positions to t+δt:

(24) $$r\left(t+\delta t\right)=r\left(t\right)+v\left(t+\frac{\delta t}{2}\right)\delta t$$

(8) Return to step (1) and repeat above steps for the next time step.

### 2.4. Replica-Exchanging Self-Guided Langevin Dynamics (RXSGLD)

Replica-exchanging molecular dynamics (REMD) utilizes high temperatures to overcome energy barriers to achieve enhanced sampling. It requires significant overlaps in conformational distributions between exchanging replicas. Because conformational distributions are very sensitive to temperatures, for large systems, to perform a REMD of a typical temperature range, many replicas are needed to produce statistically meaningful overlaps. The number of replicas needed increases exponentially with the system size, meaning that REMD are difficult to apply for large systems due many replicas being needed. Unlike high-temperature simulation, SGLD only enhances low-frequency motions and causes much less perturbation to simulation systems. Therefore, replicas with different guiding factors have large overlap in conformational distribution and much fewer replicas are needed to reach the same enhancement as REMD. To contrast with RXSGLD, we call high-temperature replica-exchanging Langevin dynamics TRXLD.

The RXSGLD method [26] published in 2012 was based on the old SGLD partition function [7]:

(25) $$\Theta_{\mathrm{SGLD}}\approx \sum \exp\left(-\frac{\lambda_{\mathrm{lf}}\chi_{\mathrm{lf}}{\tilde{E}}_{p}}{kT}-\frac{\lambda_{\mathrm{hf}}\chi_{\mathrm{hf}}(E_{p}-{\tilde{E}}_{p})}{kT}\right)$$

Here, we introduce frequency-separated factors to account for contributions from low- and high-frequency motions. $\lambda_{\mathrm{lf}}$ and $\lambda_{\mathrm{hf}}$ are the low-frequency energy factor and the high-frequency energy factor, respectively. They are the average projections of the total forces in the direction of the interaction forces:

(26) $$\lambda_{\mathrm{lf}}=\frac{\left\langle \sum_{i}({\tilde{f}}_{i}+{\tilde{g}}_{i}-\gamma_{i}{\tilde{p}}_{i}){\tilde{f}}_{i}\right\rangle }{\left\langle \sum_{i}{\tilde{f}}_{i}{\tilde{f}}_{i}\right\rangle }$$

(27) $$\lambda_{\mathrm{hf}}=\frac{\left\langle \sum_{i}\left(f_{i}-{\tilde{f}}_{i}+g_{i}-{\tilde{g}}_{i}-\gamma_{i}(p_{i}-{\tilde{p}}_{i})\right)(f_{i}-{\tilde{f}}_{i})\right\rangle }{\left\langle \sum_{i}\left(f_{i}-{\tilde{f}}_{i}\right)(f_{i}-{\tilde{f}}_{i})\right\rangle }$$

$\chi_{\mathrm{lf}}$ and $\chi_{\mathrm{hf}}$ are the low-frequency collision factor and the high-frequency collision factor, respectively. They are the projections of the guiding forces in the direction of the friction forces:

(28) $$\chi_{\mathrm{lf}}=\frac{{\tilde{T}}_{0}}{\tilde{T}}=1-\frac{\left\langle \sum_{i}{\tilde{g}}_{i}\gamma_{i}{\tilde{p}}_{i}\right\rangle }{\left\langle \sum_{i}\gamma_{i}^{2}{\tilde{p}}_{i}{\tilde{p}}_{i}\right\rangle }$$

(29) $$\chi_{\mathrm{hf}}=\frac{T-{\tilde{T}}_{0}}{T-\tilde{T}}=\frac{T-\chi_{\mathrm{lf}}\tilde{T}}{T-\tilde{T}}=1-\frac{<\sum_{i}\gamma_{i}(g_{i}-{\tilde{g}}_{i})\cdot (p_{i}-{\tilde{p}}_{i})>}{<\sum_{i}\gamma_{i}^{2}(p_{i}-{\tilde{p}}_{i})\cdot (p_{i}-{\tilde{p}}_{i})>}$$

where $\tilde{T}$ is the low-frequency temperature. We can calculate $\tilde{T}$ from the low-frequency momentum:

(30) $$\tilde{T}=\frac{1}{N_{\mathrm{DF}}k}\left\langle \sum_{i}\frac{{\tilde{p}}_{i}^{2}}{m_{i}}\right\rangle$$

Here, $N_{\mathrm{DF}}$ is the degrees of freedom of the simulation system. ${\tilde{T}}_{0}$ is the low-frequency temperature when no guiding forces are applied.

The conformational search ability of an SGLD simulation can be quantitatively described by the self-guiding temperature defined as follows:

(31) $$T_{\mathrm{SG}}=\frac{\chi_{\mathrm{hf}}}{\chi_{\mathrm{lf}}}T=\frac{\tilde{T}(T-{\tilde{T}}_{0})}{{\tilde{T}}_{0}(T-\tilde{T})}T$$

**Figure 1:**
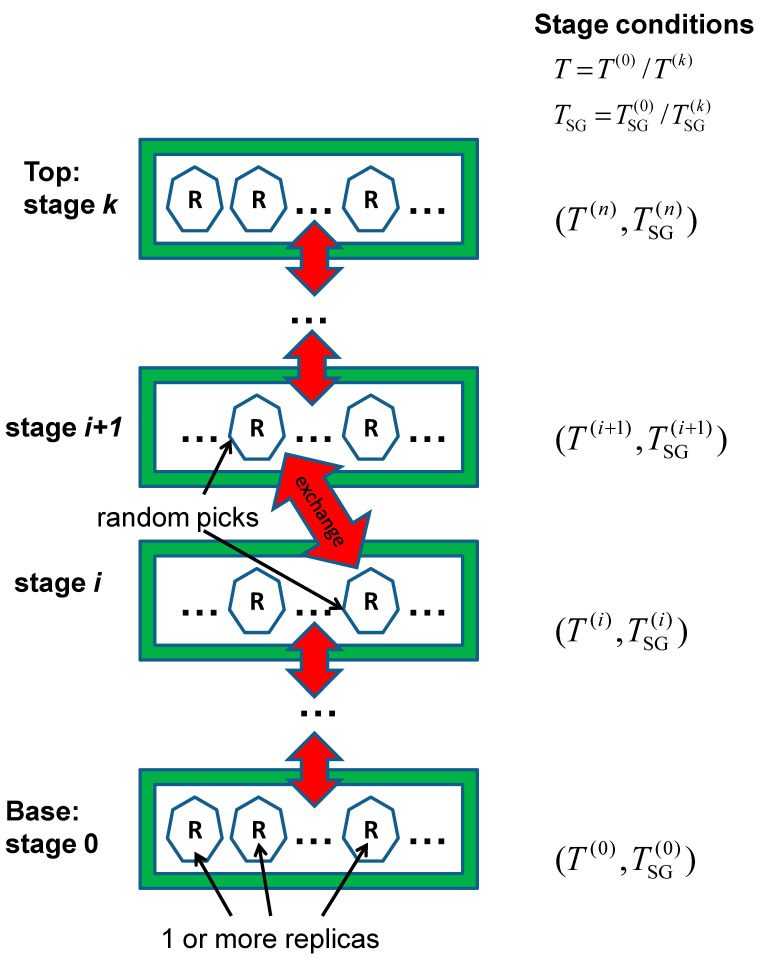
Illustration of the replica-exchanging self-guided Langevin dynamics simulation (RXSGLD). Stages are shown with green boxes. The simulation conditions, $T^{\left(0\right)}$ and $T_{\mathrm{SG}}^{\left(i\right)}$, for each stage are labelled beside its green box. A simulation system is replicated to many copies, called replicas. Replicas are drawn as hexagons inside each green box. There are one or more replicas on each stage. Replicas between neighbouring stages can exchange(marked with red double headed arrows). The base stage has the simulation condition of interest, $T^{\left(0\right)}$ and $T_{\mathrm{SG}}^{\left(0\right)}=T^{\left(0\right)}$. In TRXLD simulation, each replica undergoes an LD simulation, and different stages have different temperatures, $T^{\left(i\right)}\geq T^{\left(0\right)}$. In an RXSGLD simulation, each replica undergoes a SGLD simulation and different stages have different self-guiding temperatures, $T_{\mathrm{SG}}^{\left(i\right)}\geq T^{\left(0\right)}$.

An RXSGLD simulation is illustrated by the scheme shown in Figure 1. Each simulation condition defines a stage, and there are *k* + 1 stages with different simulation conditions. Stage 0 is set to have the condition of interest ($T_{\mathrm{SG}}^{\left(0\right)}=T$ and $T^{\left(0\right)}=T$) and is termed the base stage. We set different guiding temperatures, $T_{\mathrm{SG}}^{\left(i\right)}$, and the same or different temperatures, $T^{\left(i\right)},\;for\;the\;other\;k\;stages$. The top stage with $T^{\left(k\right)}$ and $T_{\mathrm{SG}}^{\left(k\right)}$ has the maximum conformational searching ability.

On each stage, there are one or more replicas of the simulation system, as shown in Figure 1. The number of replicas on each stage can be different from stage to stage. Replica exchanges between stages are performed by randomly choosing a pair of replicas in different stages and exchanging their stages according to the exchange probability.

The exchange probability is the key to maintaining correct ensemble distributions on each stage. From the SGLD partition function, Equation (25), we can derive the exchange probability between stages. On stage *m*, conformation *i* and temperature are denoted as $X_{m}^{\left(i\right)}$ and $T_{m}$. From Equation (25), we have the SGLD distribution probability of $X_{m}^{\left(i\right)}$:

(32) $$\rho_{\mathrm{SGLD}}(X_{m}^{\left(i\right)})=\frac{1}{\Theta_{\mathrm{SGLD}}^{\left(m\right)}}\exp\left(-\frac{\lambda_{\mathrm{lf}}^{\left(m\right)}\chi_{\mathrm{lf}}^{\left(m\right)}{\tilde{E}}_{p}^{\left(i\right)}}{kT_{m}}-\frac{\lambda_{\mathrm{hf}}^{\left(m\right)}\chi_{\mathrm{hf}}^{\left(m\right)}(E_{p}^{\left(i\right)}-{\tilde{E}}_{p}^{\left(i\right)})}{kT_{m}}\right)=\frac{1}{\Theta_{\mathrm{SGLD}}^{\left(m\right)}}\exp\left(-l_{m}{\tilde{E}}_{p}^{\left(i\right)}-h_{m}E_{p}^{\left(i\right)}\right)$$

Here, the parameters are defined as following:

(33) $$l_{m}=\frac{\lambda_{\mathrm{lf}}^{\left(m\right)}\chi_{\mathrm{lf}}^{\left(m\right)}-\lambda_{\mathrm{hf}}^{\left(m\right)}\chi_{\mathrm{hf}}^{\left(m\right)}}{kT_{m}}$$

and

(34) $$h_{m}=\frac{\lambda_{\mathrm{hf}}^{\left(m\right)}\chi_{\mathrm{hf}}^{\left(m\right)}}{kT_{m}}$$

A replica exchange of a pair of replicas, $X_{m}^{\left(i\right)}$ and $X_{n}^{\left(j\right)}$, changes the state from ${\{\ldots ,X}_{m}^{\left[i\right]},\ldots ,X_{n}^{\left[j\right]},\ldots \}$ to ${\{\ldots ,X}_{m}^{\left[j\right]},\ldots ,X_{n}^{\left[i\right]},\ldots \}$. The exchange probability, $\pi_{\mathrm{RX}}$, has the following form.

(35) $$\begin{array}{l} \pi_{\mathrm{RX}}\left(\left\{X_{m}^{\left[i\right]},X_{n}^{\left[j\right]}\}\to \{X_{m}^{\left[j\right]},X_{n}^{\left[i\right]}\right\}\right)=\frac{\rho_{\mathrm{SGLD}}\left(X_{m}^{\left[j\right]}\right)\rho_{\mathrm{SGLD}}\left(X_{n}^{\left[i\right]}\right)}{\rho_{\mathrm{SGLD}}\left(X_{m}^{\left[i\right]}\right)\rho_{\mathrm{SGLD}}\left(X_{n}^{\left[j\right]}\right)}\\ \approx \exp\left(-\left(l_{m}-l_{n}\right)\left({\tilde{E}}_{p}(X_{n}^{\left[j\right]})-{\tilde{E}}_{p}(X_{m}^{\left[i\right]})\right)-\left(h_{m}-h_{n}\right)\left(E_{p}(X_{n}^{\left[j\right]})-E_{p}(X_{m}^{\left[i\right]})\right)\right) \end{array}$$

Here, we approximate the following: ${\tilde{E}}_{p}(X_{m}^{\left[j\right]})\approx {\tilde{E}}_{p}(X_{n}^{\left[j\right]})$, and ${\tilde{E}}_{p}(X_{n}^{\left[i\right]})\approx {\tilde{E}}_{p}(X_{m}^{\left[i\right]})$. When $T_{m}=T_{n},$ the low-frequency energies at different stages are the same for the same conformation, and this approximation is accurate. For RXSGLD simulations, we recommend having the same temperature on all stages.

The low-frequency exchange coefficient, $l_{m}=\beta_{m}\left(\lambda_{\mathrm{lf}}^{\left(m\right)}\chi_{\mathrm{lf}}^{\left(m\right)}-\lambda_{\mathrm{hf}}^{\left(m\right)}\chi_{\mathrm{hf}}^{\left(m\right)}\right)$, and the high-frequency exchange coefficient, $h_{m}=\beta_{m}\lambda_{\mathrm{hf}}^{\left(m\right)}\chi_{\mathrm{hf}}^{\left(m\right)}$, are needed to evaluate the exchange probability in an RXSGLD simulation, which is calculated from parameters $\lambda_{\mathrm{lf}}^{\left(m\right)}$, $\lambda_{\mathrm{hf}}^{\left(m\right)}$, $\chi_{\mathrm{lf}}^{\left(m\right)}$, and $\chi_{\mathrm{hf}}^{\left(m\right)}$, calculated during the simulations.

It should be noted that the RXSGLD method presented above is based on the old SGLD partition function [7]. The generalized SGLD method [10] developed recently has a new form of partition function, as shown in Equation (6). With this new partition function, it is straightforward to design replica exchange simulations with varying guiding factors and/or temperatures [26]. The stage *m* is defined by $\left\{\lambda_{m},\mu_{m},\beta_{m}\right\}$, containing the guiding factors $\lambda_{m},\mu_{m}$, and temperature factor $\beta_{m}$. The conformation *i* at stage *m* is denoted as ${Χ}_{m}^{\left(i\right)}$.

The exchange probability, $\pi_{\mathrm{RX}}$, for two replicas exchanging between stages *m* and *n*, has the following form.

(36) $$\begin{array}{l} \pi_{\mathrm{RX}}\left(\left\{X_{m}^{\left[i\right]},X_{n}^{\left[j\right]}\}\to \{X_{m}^{\left[j\right]},X_{n}^{\left[i\right]}\right\}\right)=\frac{\rho_{\mathrm{SGLD}}\left(X_{m}^{\left[j\right]}\right)\rho_{\mathrm{SGLD}}\left(X_{n}^{\left[i\right]}\right)}{\rho_{\mathrm{SGLD}}\left(X_{m}^{\left[i\right]}\right)\rho_{\mathrm{SGLD}}\left(X_{n}^{\left[j\right]}\right)}\\ =\exp\left(\left(\beta_{m}-\beta_{n}\right)\left(E_{P}^{\left(i\right)}-E_{P}^{\left(j\right)}\right)+(\beta_{m}\left(\mu_{m}-{\hat{\mu }}_{m}\right)-\beta_{n}\left(\mu_{n}-{\hat{\mu }}_{n}\right))\left({\tilde{E}}_{P}^{\left(i\right)}-{\tilde{\tilde{E}}}_{P}^{\left(i\right)}-{\tilde{E}}_{P}^{\left(j\right)}-{\tilde{\tilde{E}}}_{P}^{\left(j\right)}\right)\right) \end{array}$$

Equation (36) is much simpler than Equation (35) and removes the need to calculate parameters $\lambda_{\mathrm{lf}}^{\left(m\right)}$, $\lambda_{\mathrm{hf}}^{\left(m\right)}$, $\chi_{\mathrm{lf}}^{\left(m\right)}$, and $\chi_{\mathrm{hf}}^{\left(m\right)}$. A development along this route will be addressed in future.

### 2.5. Self-Guided Langevin Dynamics via Generalized Langevin Equation

Development of SGLD is based on the understanding that low-frequency motion need be increased to enhance conformational search. This idea has been questioned, regarding its theoretical basis. Exploring stochastic dynamics, we found that a SGLD-like equation of motion can be derived from the generalized Langevin equation (GLE) [10]. GLE has the following form:

(37) $${\dot{p}}_{i}=f_{i}-\gamma \int_{-\infty }^{t}d\tau Κ(t-\tau )p_{i}(\tau )+\eta_{i}$$

In Equation (37), Κ(t−τ) is a memory kernel, which can take a variety of forms. $\eta_{i}(t)$ is a zero-mean Gaussian noise, whose covariance is related to the kernel, Κ(t−τ), according to the fluctuation–dissipation theorem:

(38) $$<\eta_{i}(t)\eta_{j}(t^{\prime })>=\delta_{ij}m_{i}kT\gamma Κ(t-t^{\prime })$$

The memory kernel, Κ(t−τ), can take many forms. If we choose a memory kernel of the following form:

(39) $$Κ(t)=2\delta (t)-\frac{\lambda }{t_{L}}e^{-\frac{t}{t_{L}}}$$

with the following convention:

(40) $$\int_{-\infty }^{t}\delta (t-\tau )P(\tau )d\tau =\frac{1}{2}P(t)$$

We obtain the following:

(41) $$\int_{-\infty }^{t}d\tau Κ\left(t-\tau \right)p_{i}\left(\tau \right)=p_{i}-\lambda {\tilde{p}}_{i}$$

The noise term must be consistent with this kernel to satisfy the following expression:

(42) $$\eta_{i}(t)=R_{i}(t)-\frac{\upsilon }{t_{L}}\int_{-\infty }^{t}R_{i}(\tau )e^{-\frac{\tau }{t_{L}}}d\tau =R_{i}(t)-\nu {\tilde{R}}_{i}(t)$$

Here, parameter υ≥0 is a parameter related to λ. Therefore, the GLE is as follows:

(43) $${\dot{p}}_{i}=f_{i}-\gamma p_{i}+\lambda \gamma {\tilde{p}}_{i}+R_{i}-\nu {\tilde{R}}_{i}(t)$$

Equation (43) resembles the SGLD equation of motion [6] with an additional term of local average random forces, $-\nu {\tilde{R}}_{i}(t)$. We call Equation (43) the equation of motion for SGLD-GLE. It is easy to observe the following:

(44) $$\begin{array}{l} Κ\left(t-t^{\prime }\right)\equiv \frac{<\eta_{i}\left(t\right)\eta_{i}\left(t^{\prime }\right)>}{m_{i}kT\gamma }\\ =\frac{1}{m_{i}kT\gamma }(<R_{i}\left(t\right)R_{i}\left(t^{\prime }\right)>-\frac{\nu }{t_{L}}\int_{-\infty }^{t}<R_{i}\left(\tau \right)R_{i}\left(t^{\prime }\right)>e^{-\frac{t^{\prime }-\tau }{t_{L}}}d\tau >\\ -\frac{\nu }{t_{L}}\int_{-\infty }^{t^{\prime }}<R_{i}\left(t\right)R_{i}\left(\tau^{\prime }\right)>e^{-\frac{t^{\prime }-\tau^{\prime }}{t_{L}}}d\tau^{\prime }+\frac{\nu^{2}}{t_{L}^{2}}\int_{-\infty }^{t}\int_{-\infty }^{t^{\prime }}<R_{i}\left(\tau \right)R_{i}\left(\tau^{\prime }\right)>e^{-\frac{\left(t^{\prime }-\tau^{\prime }\right)-\left(t^{\prime }-\tau^{\prime }\right)}{t_{L}}}d\tau^{\prime }d\tau )\\ =2\delta \left(t-t^{\prime }\right)-\frac{2\nu }{t_{L}}\int_{-\infty }^{t}\delta \left(t^{\prime }-\tau \right)e^{-\frac{t^{\prime }-\tau }{t_{L}}}d\tau -\frac{2\nu }{t_{L}}\int_{-\infty }^{t^{\prime }}\delta \left(t-\tau^{\prime }\right)e^{-\frac{t^{\prime }-\tau^{\prime }}{t_{L}}}d\tau^{\prime }\\ \;+\frac{2\nu^{2}}{t_{L}^{2}}\int_{-\infty }^{t}\int_{-\infty }^{t^{\prime }}\delta \left(\tau -\tau^{\prime }\right)e^{-\frac{\left(t^{\prime }-\tau^{\prime }\right)-\left(t^{\prime }-\tau^{\prime }\right)}{t_{L}}}d\tau^{\prime }d\tau \\ \;=2\delta (t-t^{\prime }\;)-\frac{\nu (2-\nu )}{t_{L}}e^{-\frac{\left|t-t^{\prime }\right|}{t_{L}}} \end{array}$$

which becomes Equation (39) when the following is true:

(45) λ=ν(2−ν)

Equation (45) has two roots, $\nu =1\pm \sqrt{1-\lambda }$. Both roots are statistically equivalent and can therefore be used interchangeably. For convenience, we use $\nu =1-\sqrt{1-\lambda }$. SGLD-GLE satisfies the detailed balance principle and can exactly preserve the canonical ensemble [9].

## 3. Example Simulations

SGMD/SGLD simulations are setup using three parameters, the local averaging time, $t_{L}$, the momentum guiding factor, λ, and/or the force guiding factor, μ. $t_{L}$ defines the frequency threshold, ${1/t}_{L}$, meaning that motions with frequency higher than this threshold are more likely to be filtered out. Typically, we set $t_{L}=0.2\;ps$ to filter out covalent bonding vibrations. Larger $t_{L}$ can be used if slower motions are to be enhanced. λ=−1~1 can be used, which corresponds to μ=0.3177~−0.3247.

### 3.1. SGLD Simulation of a Skewed Double Well System

To illustrate the enhancement in energy barrier crossing and the reweighting of conformational distribution, we designed a skewed double well (SDW) potential of the following form:

(46) $$\varepsilon \left(x,y,z\right)=ay^{2}(y^{2}-{2y}_{0}^{2})+b{\left(y+y_{0}\right)}^{2}+c\left(x^{2}+z^{2}\right)$$

**Figure 2:**
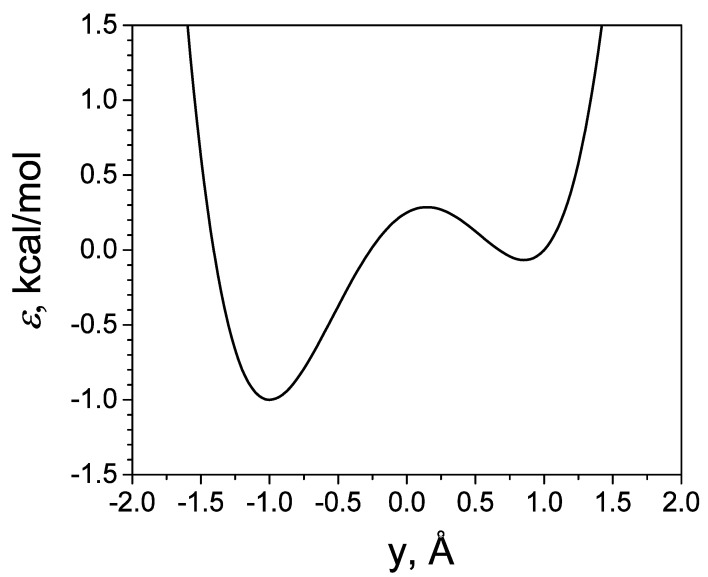
The potential profile of the skewed double well along the y-axis at x=0 and z=0. The potential parameters are a = 1 kcal/mol, b = 0.25 kcal/mol, c = 1000 kcal/mol, and $y_{0}=1\;Å$.

The energy surface defined by Equation (46) has two wells, at $y\approx -y_{0}\;$ and $y\approx y_{0}$, respectively. The well depth is defined by *a*. *b* is the skew parameter, which defines the energy difference between the two wells. *c* is the restrict parameter in *x* and *z* dimensions. Figure 2 shows the energy profile along the y axis at x=0 and z=0. Here, we set $y_{0}=1Å$, *a* = 1 kcal/mol, *b* = 0.25 kcal/mol, and *c* = 1000 kcal/mol. The large *c* restricts the energy surface narrowly in the *x* and *z* directions, so that the movement in *x* and *z* dimensions are high-frequency motions.

**Figure 3:**
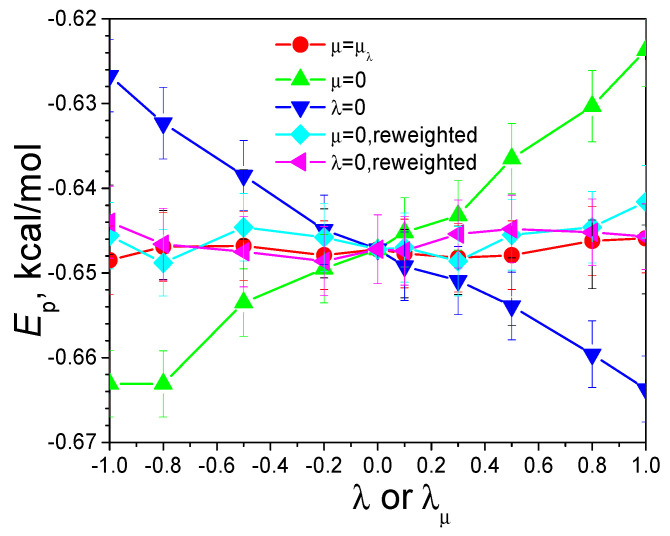
Guiding factor dependence of the average potential energies of the SDW system. For SGLD simulations with λ=0, the *x*-axis uses the $\lambda_{\mu }$ values converted from μ according to Equation (4). Reweighting is performed according to Equation (7).

The simulation is carried out with an argon atom on the SDW energy surface. A friction constant of 10/ps is applied. SGLD simulations with various guiding factors are carried out for a length of 100 ns. The local average time, $t_{L}$, is set to 0.2 ps. Figure 3 plots the average potential energies of the SDW system obtained in these simulations. As can be seen, the potential energy increases with λ. This is because a larger λ results in an enhanced low-frequency motion and more high-energy states to be sampled. On the other hand, the potential energy decreases with μ or $\lambda_{\mu }$. This is because a larger μ causes more of the low potential energy region to be sampled. When ${\mu =\lambda }_{\mu }$, we can see that average energies remain almost constant from λ=−1 to λ=1. This result demonstrates that the bias effects from both guiding forces cancel each other out when balanced guiding factors are used.

The reweighting factor calculated through Equation (7) can quantitatively describe the bias effects of the guiding forces. Figure 3 also shows the reweighted averages from the SGLD simulations. As can be seen from Figure 3, the reweighted averages agree well with the canonical averages from the LD (λ=0, μ=0) simulation. These reweighting results support that the approximation of ensemble averages with long-time local averages in the estimation of apparent friction constants is valid.

**Figure 4:**
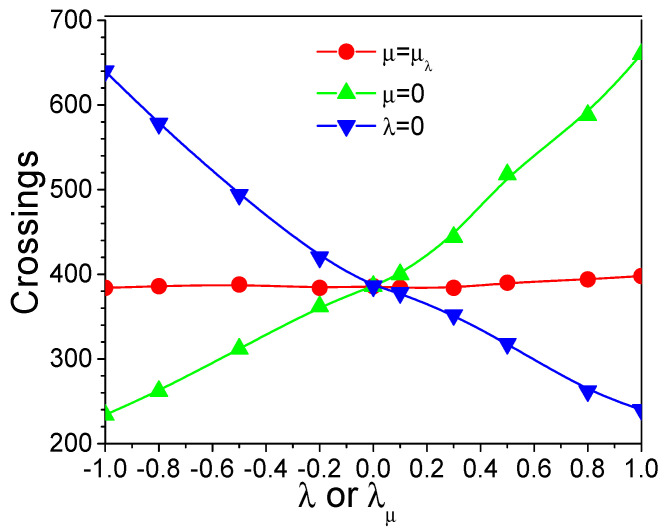
Energy barrier crossings of the SDW system at different guiding factors. *x*-axis is λ or $\lambda_{\mu }$ values converted from μ according to Equation (4). *y*-axis is the number of crossings.

The static energy barrier on the SDW potential surface provides a convenient way to examine the energy barrier crossing ability. Figure 4 shows the number of energy barrier crossings during the SGLD simulations. We can see that the crossing numbers change almost proportionally with the guiding factors. An increase in the momentum guiding factor enhances more in the low-frequency motion and increases the power to overcome energy barriers. A negative force guiding factor results in a lower energy barrier, so that crossing energy barrier becomes easier. The opposite effects of the two guiding factors can cancel each other out when ${\lambda =\lambda }_{\mu }$. These simulation results show that either a positive λ or a negative μ can be used to enhance energy barrier crossing.

### 3.2. SGMD Simulation of Liquid Argon

We further illustrate enhanced sampling of SGMD with an argon fluid under periodic boundary condition. The fluid argon system has 500 argon atoms in a cubic box of 28.53 × 28.53 × 28.53 Å^3^. The Lennard-Jones 6–12 potential with ε = 119.8 K and σ = 3.405 Å is used to calculate interaction between argon atoms. The isotropic periodic sum (IPS) method [31] is used to calculate long-range contributions with an IPS radius or cutoff distance of 10 Å. All simulations are carried out for 10 ns in NVE ensemble, with the temperature set to 100 K. For SGMD simulations, the local average time is set to $t_{L}=0.2\;ps$.

**Figure 5:**
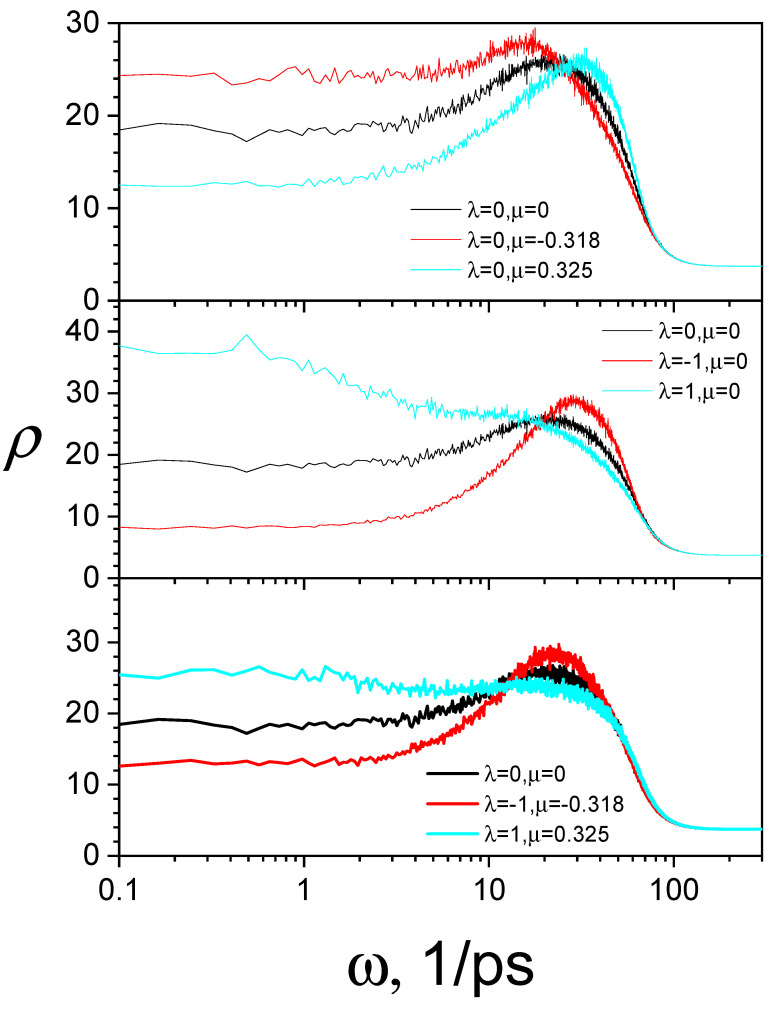
Comparison of the spectrums of the argon fluid from the SGMD simulations with different guiding factors. The guiding factors are labelled on each panel. Top panel: varying force guiding factors, middle panel: varying momentum guiding factors; bottom panel: varying balanced guiding factors.

To examine the frequency distribution of atomic motion, we calculate the power spectrums from these simulations. Figure 5 shows the power spectrums of the argon fluid at different guiding factors. The top panel of Figure 5 compares the power spectrums of SGMD simulations at different force guiding factors. We can see that when the force guiding factor changes from negative to positive, the spectrum’s high-frequency part goes up, while the spectrum’s low-frequency part goes down. This means that the force guiding force suppresses the low-frequency motion and enhances the high-frequency motion. In other words, a negative force guiding factor promotes the low-frequency motion. The middle panel of Figure 5 compares the power spectrums at different momentum guiding factors. As can be seen, when the momentum guiding factor changes from negative to positive, the low-frequency portion goes up and the high-frequency portion goes down, indicating that the momentum guiding force enhances the low-frequency motion and suppresses the high-frequency motion, opposite to the force guiding force.

The bottom panel compares the spectrums from the SGMD simulations with balanced guiding factors. Again, we see that, the high-frequency portion goes down and the low-frequency portion goes up when the momentum guiding factor changes from negative to positive, like the cases with varying momentum guiding factors. It is clear from Figure 5 that the guiding factors alter molecular motion in a frequency-dependent way.

The spectrum at 0 frequency represents the diffusion constant. Clearly, a positive momentum guiding factor or a negative force guiding factor will result in accelerated diffusion. For macromolecular systems, the low-frequency spectrum represents the conformational change speed. We can expect a positive momentum guiding factor or a negative force guiding factor will result in accelerated conformational search.

### 3.3. RXSGLD Simulation of β-Hairpin Folding

Temperature-based replica-exchange simulation has been widely used in enhanced conformational search. However, this method is difficult with large systems. The ratio of successful replica exchange depends on the overlap of conformational distributions between replicas. The larger a system is, the smaller the overlap in conformational distributions between replicas. To reach similar exchange ratio, the temperature difference must be reduced, which increases the number of replicas to achieve the same temperature range. In other words, the temperature-based replica-exchange method is not size-extensive. For a successful temperature-based replica-exchange simulation, a replica needs to travel through all temperature states many times. These temperature states are called the stages, and the travel of replicas through all stages is called replica diffusion. The number of stages increases exponentially with the system size. A large number of stages will slow down replica diffusion from the top stage to the base stage, which in turn reduces the conformational sampling efficiency.

Protein folding in explicit water is a challenging application of replica-exchange simulations due to its large size and long time-scale. RXSGLD is a suitable approach for this type of study, due to the enhanced sampling of SGLD and the small number of stages for replica exchange. An aqueous solution of a nine-residue β-hairpin folding peptide is a good example to demonstrate the application of RXSGLD in large systems. This nine-residue peptide was designed by Blanco et al. [32] and was modified from the β-hairpin of α-amylase inhibitor tendamistat (residues 15–23). This peptide has an amino acid sequence of Tyr(1)-Gln(2)-Asn(3)-Pro(4)-Asp(5)-Gly(6)-Ser(7)-Gln(8)-Ala(9). A cubic box of 829 TIP3P [33] water molecules are used to solvate the peptide, and a sodium ion is placed in the box to neutralize the system. The cubic box is 30 × 30 × 30 Å^3^ in size. The temperature is maintained with a Langevin heat bath with a collision frequency of 1/ps. A CHARMM 22 force field [34] is used for energy calculation and a 3D isotropic periodic sum (IPS) method is used for long-range nonbonded interaction calculation. The local region radius of 10 Å is used for 3D IPS calculation of electrostatic and Lennard–Jones energies [31,35,36]. The temperature replica exchange Langevin dynamics (TRXLD) simulations have eight temperature stages. Three TRXLD simulations are performed with temperature (*T*) ranges of 274/310 K, 274/350 K, and 274/400 K. The RXSGLD simulations have eight stages with the self-guiding temperature (*T*_SG_) ranges of 274/310 K, 274/350 K, and 274/400 K, but temperature is the same for all stages, *T* = 274 K. The initial conformation of the peptide for all simulations is a fully extended conformation. In all replica-exchange simulations, every replica is simulated for 20 ns.

**Figure 6:**
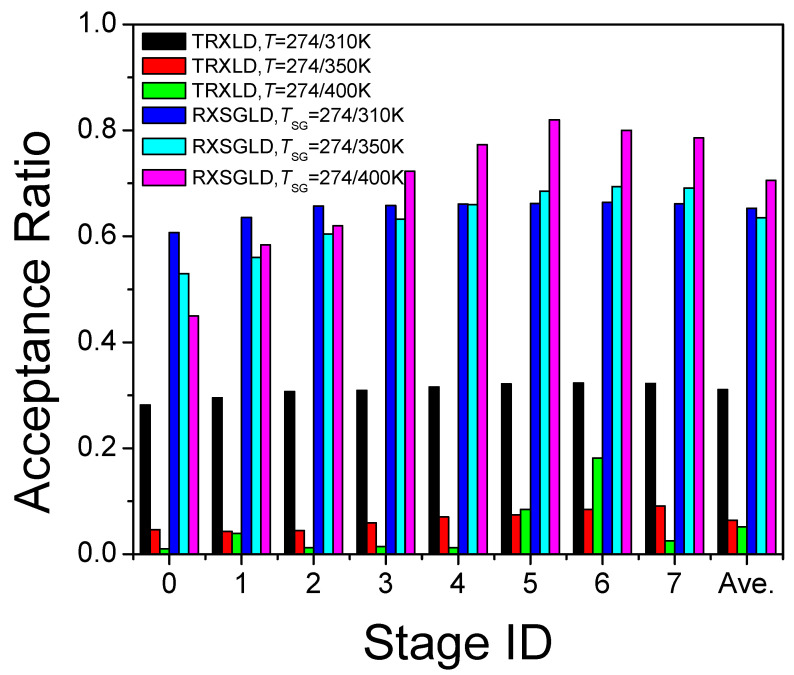
Comparison of exchange acceptance ratios at each stage in the TRXLD and RXSGLD simulations. The simulation system is the nine-residue β-hairpin folding peptide solvated in a cubic box of 829 TIP3P water molecules.

The replica-exchange acceptance ratio is a criterion of conformational searching efficiency. Figure 6 compares the acceptance ratios on each stage in these simulations. For the TRXLD simulation with a small temperature range, T = 274/310 K, the average exchange acceptance ratio is 31.1%, which is acceptable. However, if temperature ranges become more reasonable, T = 274/350 K and T = 274/400 K, the average exchange acceptance ratios reduce to 6.4% and 5.2%, respectively. With such a low acceptance ratio, replicas remain in the same stage most of the time and conformational search at high temperature is difficult to pass through. This low acceptance ratio is due to the large size of the simulation system. The larger the system size, the lower the acceptance ratio in TRXLD simulations. Therefore, TRXLD is limited by system sizes.

For all three RXSGLD simulations, the exchange acceptance ratios are remarkably high (65.3%, 63.5%, and 70.2%). RXSGLD relies on large guiding factors to achieve enhanced conformational sampling. The guiding forces only promote low-frequency motion, because low-frequency motions account for a very small portion of thermal motion, and large guiding factors do not change conformational distribution as much as temperature. Therefore, replicas with different guiding factors have a high exchange acceptance ratio.

**Figure 7:**
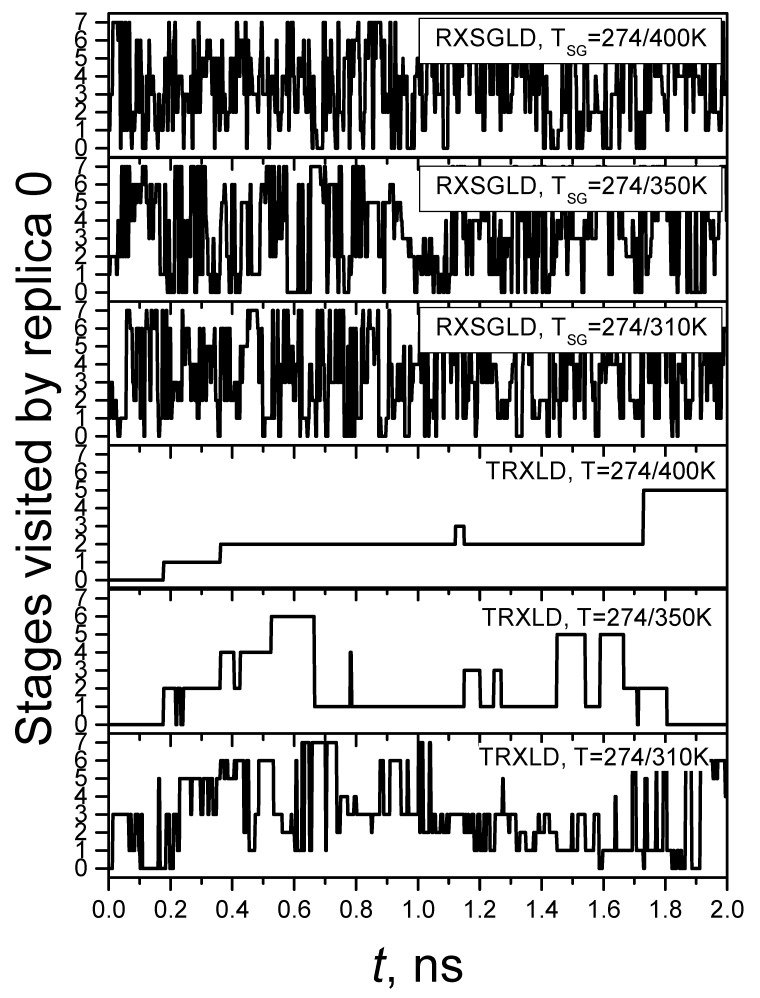
Diffusion trajectories of replica 0 in the TRXLD and RXSGLD simulations. These simulations have eight stages. The simulation system is the nine-residue β-hairpin folding peptide solvated in a cubic box of 829 TIP3P water molecules.

The efficiency of replica-exchanging simulations can also be examined through the diffusion of replicas crossing the stages. Replica diffusion trajectories crossing the stages provide a direct picture of how efficiently the sampling information transfers. Figure 7 shows the diffusion trajectory of replica 0 during the simulations. For TRXLD with T = 274/310 K, it takes replica 0 more than 0.6 ns to reach stage 7, while for TRXLD simulations with T = 274/350 K and T = 274/400 K, replica 0 takes 2.35 ns and 3.28 ns (beyond the plotting range), respectively, to reach stage 7. In all three RXSGLD simulations, it takes less than 0.1 ns for replica 0 to reach stage 7. These results demonstrate that RXSGLD is much more efficient than TRXLD.

**Figure 8:**
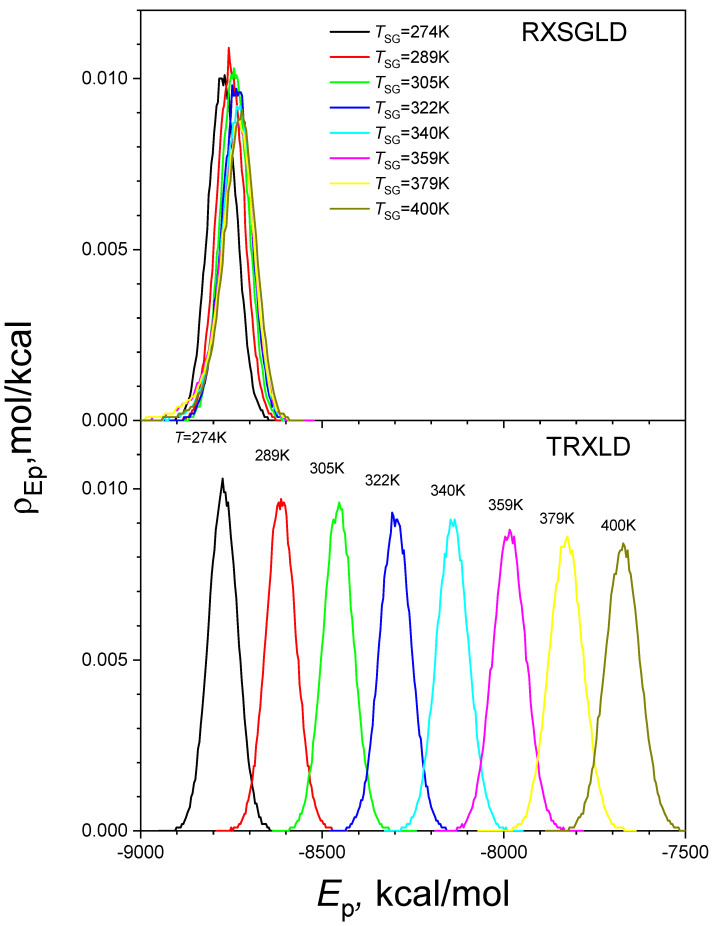
Potential energy distributions at each stage in the TRXLD (T=274/400 K) and RXSGLD ($T_{\mathrm{SG}}=274/400\;K$ and $T=T_{0}=274\;K$) simulations. The simulation system is the nine-residue β-hairpin folding peptide solvated in a cubic box of 829 TIP3P water molecules.

Figure 8 shows the potential energy distributions at the eight stages of the TRXLD simulation with *T* = 274/400 K and of the RXSGLD simulations with *T*_SG_ = 274/400 K. Clearly, the energy distributions in the TRXLD simulation are very different between stages, and there are very small overlaps between neighbouring stages. Remarkably, the stage energy distributions in the RXSGLD simulation are very close to one other and have significant overlap with each other. This large overlap in stage energies makes the acceptance ratio high in RXSGLD simulations.

**Figure 9:**
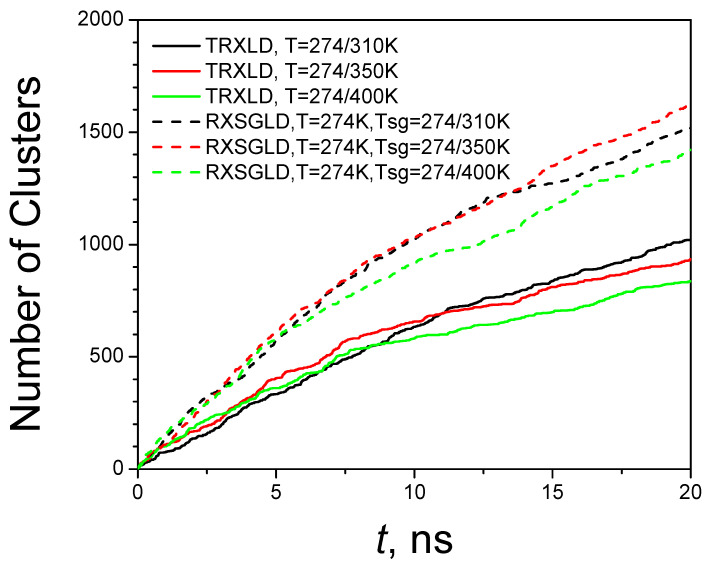
Conformational clusters reached during the TRXLD and RXSGLD simulations. The simulation system is the nine-residue β-hairpin folding peptide solvated in a cubic box of 829 TIP3P water molecules.

Because the conformation space of a protein is huge, it is not expected to reach all the important conformational states in a simulation. Simulation trajectories from different initial velocities are very likely to visit different conformations, even in the same simulation condition. One way to measure the progress of conformational search is by examining the conformational clusters. We can compare the conformational searching abilities by examining the conformational clusters searched during the simulations. To identify conformational clusters, we propose a subset indexing clustering (SIC) method [26]. Using the SIC method, we clustered the conformations at the base stage of the TRXLD and RXSGLD simulations, and the results are shown in Figure 9. All RXSGLD simulations show significantly more clusters than the TRXLD simulations. These results again demonstrate that RXSGLD has stronger conformational searching ability than TRXLD. It is noted that the TRXLD and RXSGLD simulations with temperature range 274/400 K find even fewer clusters than simulations with a temperature range of 274/310 K after a certain time. This is because larger temperature ranges also reduce the acceptance ratio of exchange. Higher temperature causes an increase in potential energies and makes the conformation less likely to exchange with lower temperatures.

To summarise, the main difference between replica-exchanging molecular dynamics (REMD or TRXLD) and RXSGLD is the exchanging state variables. In REMD, replicas are different according to temperature, while in RXSGLD, replicas are all in the same state but undergo SGMD/SGLD simulations with different guiding parameters. In REMD, replicas have very different conformational distribution due to all motion modes changing with temperature. The significant change in conformational distribution makes replica exchange difficult, or many replicas are needed to reach a temperature high enough to have a proper sampling. In RXSGLD, different guiding parameters change only the slow motion, meaning that conformational distributions have significant overlap and replica exchange has a high successful ratio. Therefore, RXSGLD is highly recommended, especially for large systems, where conformational distributions at different temperatures have little overlap.

### 3.4. SGLD-GLE Simulation of Liquid Argon

The SGLD-GLE simulation method has a vigorous theoretical basis. Therefore, it is interesting to examine its enhancement in conformational searching ability and accuracy in sampling the canonical ensemble. We chose an argon fluid to examine the behaviour of SGLD-GLE in the *NPT* ensemble. Argon atoms interact with a Lennard–Jones potential, as described in Section 3.2. The simulation system contained 500 argon atoms, and a cubic periodic box (28.53 × 28.53 × 28.53 Å^3^) was used to contain the system. The Langevin thermal bath had a collision frequency of 10 ps^−1^. All simulations reported here used a time step of 1 fs and are 10 ns in length, and were carried out at a constant temperature of 100 K and a constant pressure of 1 atm. For comparison, LD simulations at 100 K and 130 K were also performed. Coordinates and velocities were saved every 0.05 ps for post-analysis.

**Figure 10:**
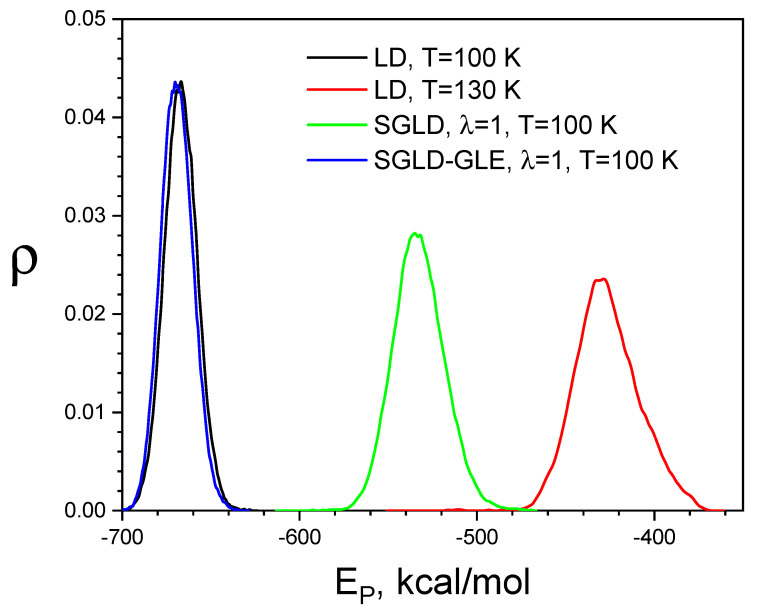
Comparison of energy distributions of the liquid argon from LD, SGLD, and SGLD-GLE simulations. All simulations were carried out in the *NPT* ensemble with *T* = 100 K, P = 1 atm, and *g* = 10/ps, except the high-temperature LD simulation, which was performed at *T* = 130 K. For both the SGLD and SGLD-GLE simulations, the guiding factor was set to λ = 1 and the local average time was set to $t_{L}=0.2\;ps$.

Figure 10 compares the conformational distributions from LD, SGLD, and SGLD-GLE simulations. As can be seen from Figure 10, the high-temperature LD and SGLD simulations sample conformations with much higher potential energies than those sampled in the low-temperature LD simulation. Comparing the distributions of LD and SGLD, we can see that SGLD elevate potential energies less than high-temperature LD. This is because SGLD only enhance low-frequency motion, while high-temperature LD elevate motion in all frequencies. The energy distribution of the SGLD-GLE simulation is almost identical to that of the LD simulation. These results demonstrate that SGLD and high temperature shift conformational distribution to the high-energy region, whereas SGLD-GLE can maintain the canonical ensemble distribution.

**Figure 11:**
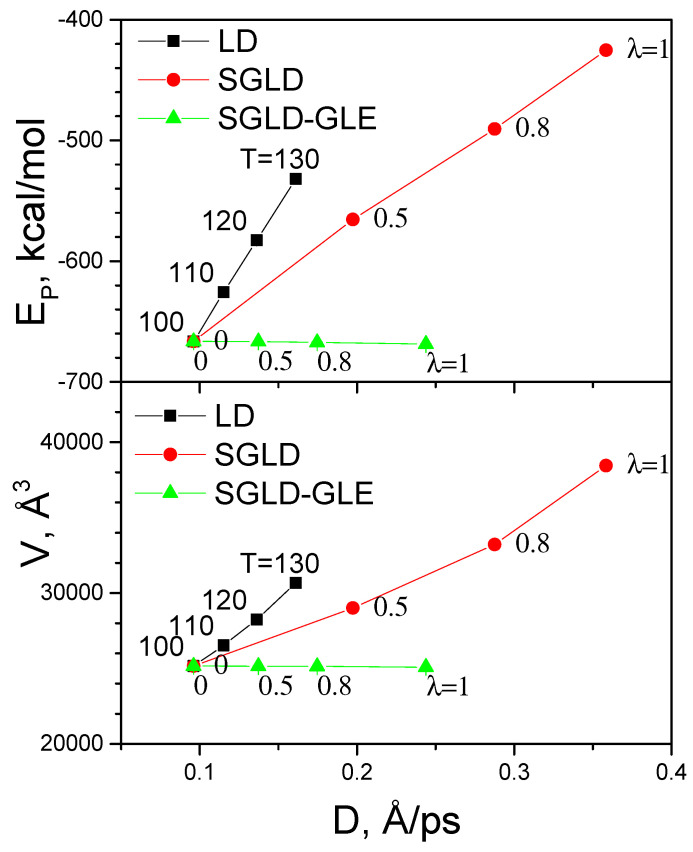
Comparison of perturbation against enhancement for the fluid argon system. The enhancement is measured by the diffusion constant. The perturbation is measured by the change in ensemble averages. The top panel is average energies vs. diffusion constants, and the bottom panel is average volumes vs. diffusion constants. High-temperature LD, SGLD, and SGLD-GLE simulations are examined. All simulations are performed in the *NPT* ensemble at *γ* = 10/ps, *T* = 100 K, and *P* = 1 atm, unless labelled otherwise. Temperatures are labelled for the high-temperature LD simulations. Guiding factors are labelled for the SGLD and SGLD-GLE simulations.

Diffusion constants represent the motion at the lowest frequency; therefore, they can be used to measure the conformational search efficiency for this homogeneous system. To quantitatively compare the enhancement in conformational sampling, we plot the average energies and average volumes as functions of the diffusion constants in these simulations (Figure 11). This plot shows how these ensemble averages change with an enhancement in conformational search. It is clear to see that temperature elevation causes the fastest energy and volume increases. To achieve similar enhancement in conformational search, as measured by the diffusion constants, SGLD requires smaller changes in average energies and volumes. Remarkably, SGLD-GLE produces almost identical average energies and volumes while achieving significantly larger diffusion constants.

**Figure 12:**
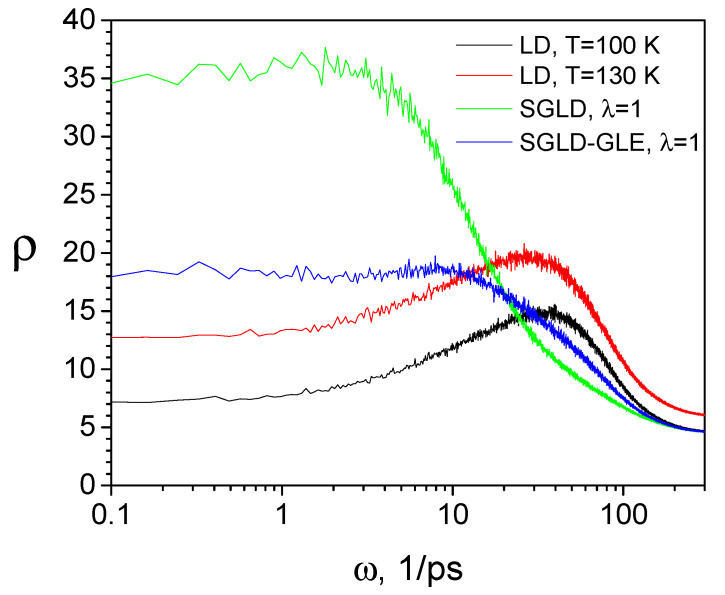
Comparison of the spectra of the argon liquid obtained from LD, SGLD, and SGLD-GLE simulations. All simulations are performed in the *NPT* ensemble with *T* = 100 K, *P* = 1 atm, and *γ* = 10/ps, except that the high-temperature LD is performed at *T* = 150 K. The guiding factor is set to λ = 1 and the local average time is set to $t_{L}=0.2\;ps$ for the SGLD and SGLD-GLE simulations.

The power spectrum of the velocity auto-correlation function is very informative regarding the dynamic property of simulated systems. The power spectrum is calculated from the following formula:

(47) $$\rho (\omega )=\int_{-\infty }^{\infty }C(t)\exp(-i\omega t)dt$$

where *C*(*t*) is the velocity autocorrelation and *ω* is the frequency. Figure 12 shows the spectra from high-temperature LD simulations and the results from SGLD and SGLD-GLE simulations. When the temperature is elevated from 100 K to 130 K, the spectrum rises at all frequencies, indicating that elevating temperature enhances all thermal motions. Comparing the spectra of SGLD and SGLD-GLE with those of LD at the same temperature (*T* = 100 K), it is clear to see that the slow motions (ω<2~3/ps) are enhanced and fast motions (ω>2~3/ps) are reduced. This threshold frequency is related to the local average time, $t_{L}$. This threshold frequency will become lower with a longer averaging time (a larger $t_{L}$). The diffusion constants, which are related to the power spectrum at 0 frequency, increase with the guiding factor, λ, in SGLD and SGLD-GLE simulations. It should be noted that SGLD shows significantly more enhancement than SGLD-GLE. This is because SGLD-GLE is restrained to maintain correct ensemble distributions. The enhancement in conformational sampling and correct ensemble distribution makes SGLD-GLE a better alternative to the conventional LD method.

## 4. Concluding Remarks

The self-guided molecular simulation methods are designed to overcome randomness in conformational search and accelerate the process to reach global minimum states. A local average scheme is the main characteristic of this method, which extracts the self-guiding information during a simulation without many overhead computing costs. The local average information is fed-back to the equation of motion to enhance low-frequency motion, which is important for conformation sampling. Two types of guiding forces, local average of momentums and local average of forces, are employed to enable a self-guided motion. The momentum-based guiding force is effective in a diffusion-controlled conformational search, while the force-based guiding force is suitable for an energy barrier-controlled conformational search.

The generalized self-guided molecular/Langevin dynamics simulation method we developed in recent years correlates the guiding factors with conformational distributions, providing quantitative understanding of the bias effects of the guiding factors on conformational distribution. This relation makes it convenient for users to choose proper guiding factors in their simulations to achieve the desired enhancement in conformational search, as well as to calculate ensemble averages through reweighting.

The progress made in understanding conformational distribution in self-guided molecular simulations allowed us to develop the replica-exchanging self-guided Langevin dynamics (RXSGLD) simulation method. This method uses SGLD to enhance conformational searching and uses replica exchange to pass information between different guiding stages. By avoiding temperature elevation, this method is suitable for large systems with high replica-exchange efficiency and can save on computing costs with a reduced number of replicas. By applying RXSGLD to the β-hairpin folding in explicit water, we demonstrated that RXSGLD has better size extensivity than REMD, and much larger systems can be effectively studied by avoiding the burden of a low exchanging ratio in REMD.

The SGLD-GLE method, developed based on the generalized Langevin equation, is an excellent replacement of the conventional LD simulation method. Our simulations show that SGLD-GLE is accurate in conformational sampling and has enhanced conformational sampling efficiency.

SGMD/SGLD has several unique characters compared to other sampling enhancement methods. We expect that SGMD/SGLD will be useful in a wide variety of simulation studies, such as phase transition, ligand docking, and protein folding.

## Data Availability

No new data were created or analyzed in this study. Data sharing is not applicable to this article.
